# Are the Reasons Why Patients Are Referred for an Orthodontic Visit Correct?

**DOI:** 10.3390/ijerph18105201

**Published:** 2021-05-13

**Authors:** Marco Di Blasio, Benedetta Vaienti, Giuseppe Pedrazzi, Diana Cassi, Marisabel Magnifico, Sara Meneghello, Alberto Di Blasio

**Affiliations:** 1 University Center of Dentistry, Department of Medicine and Surgery, University of Parma, Via Gramsci 14, 43126 Parma, Italy; benedetta.vaienti@studenti.unipr.it (B.V.); marisabel.magnifico@unipr.it (M.M.); sara.meneghello@studenti.unipr.it (S.M.); 2 Unit of Neuroscience and Interdepartmental Center of Robust Statistics (Ro.S.A.), Department of Medicine and Surgery, University of Parma, Via Gramsci 14, 43126 Parma, Italy; giuseppe.pedrazzi@unipr.it; 3 Department of Surgical, Medical, Dental and Morphological Science, University of Modena and Reggio Emilia, 41124 Modena, Italy; diana.cassi@unimore.it

**Keywords:** community dentistry, orthodontic diagnosis, delayed diagnoses, facial deformities

## Abstract

Who does refer patients for an orthodontic consultation? Which are the main reasons for the referral? Does the visit of the orthodontic specialist confirm these reasons or reveal undiagnosed problems? Is there the risk that only evident dental problems are addressed, while craniofacial malformations remain underdiagnosed? This cross-sectional epidemiologic study aims to answer these questions, analysing the clinical data collected during the orthodontic visits of 500 Caucasian young patients referred to a public health structure of northern Italy. All patients were visited by the same expert specialist in orthodontics. Clinical data were collected, analysing both dental and skeletal features. The reasons for the referral of the visit were analysed and compared with the specialistic diagnoses. In our sample, dentists, relatives/friends and paediatricians were the major source of the referrals, followed by family doctors and other facial specialists. In most cases, the reasons for the referral were dental irregularities, but approximately 80% of dental irregularities were associated with undiagnosed facial dysmorphism. Skeletal facial anomalies need an early diagnosis to prevent the development of severe facial malformations that would require invasive and expensive treatments. These findings reveal poor diagnostic skills regarding skeletal anomalies in dentists and paediatricians and the need for better specific training.

## 1. Introduction

Malocclusions, as well as dental caries and gingival diseases, are one of the most common dental problems, and the demand for orthodontic treatment is increasing in many developed countries. The subjects requiring a visit are mostly parents and doctors who care for young patients. Their main motivations are often related to problems with dental aesthetics, i.e., crowding or protruding upper teeth. Therefore, the specialist in orthodontics often identifies problems that are quite different from those that motivated the visit. In early 1971, Edgerton and Knorr [1] proposed that the motivation source was the most crucial factor in determining and predicting patient satisfaction with treatment. Proffit and White [2] have long recognized the importance of exploring a patient’s motivation for treatment at the initial consultation together with the patient’s list of treatment objectives. In their questionnaire-based study, McKiernan et al. [3] found a desire to improve dental appearance to be the primary motivating factor, followed by a wish to improve facial harmony. Similarly, Sergl and Zentner [4] studied the psychosocial aspects of patients undergoing orthodontic treatment and found that the majority of them were concerned about their poor smile aesthetics. These findings seem to be logical, as certainly the aesthetics of the smile attracts the attention of a non-specialistic observer (i.e., parents/friends). However, it would also be logical to expect attention to the entire maxillofacial complex harmony, from observers who have undergone medical training. 

The prevalence of malocclusions in a particular population was widely studied during the last two decades [5,6,7,8,9], and the results have shown wide variations, ranging from 39 to 98%. Differences in age ranges, ethnicity, social class and the number of subjects examined could explain some of the variations. Moreover, differences in the registration methods are probably the most important factors explaining these variations. Some authors [5,6,7] have evaluated occlusion using the classification developed by Björk [8] or the Angle’s [9] classification. Nowadays, several indices are available to express the orthodontic need for treatment, such as the Dental Aesthetic Index [10], the Treatment Priority Index [11], the Index of Complexity Outcome and Need [12] and the Index of Orthodontic Treatment Need [13]. Despite the extensive amount of literature on the topic, reviewed by Thilander et al. [14], few authors analysed the distribution of occlusal and dentofacial characteristics in the Italian population [15,16,17,18,19], especially in the population of northern Italy. Regardless of the method used, the specialist must carefully check not only the dental features during the orthodontic visit, but also the facial morphology which can highlight disharmonies in the growth of the maxillary bones [20]. Except for the most evident cases of facial malformation [21], these disharmonies are often more difficult to detect than the anomalies of the dentition, and it may happen that they are not identified by the person who sends the patient. The risk is that the main facial growth problems are not referred to the specialist, unless they are accompanied by aesthetic irregularities of the teeth. In this retrospective study, the authors investigated these problems and tried to answer the following questions: Who does refer patients for an orthodontic consultation? Which are the main reasons for the referral? Does the visit of the specialist in orthodontics confirm these reasons or reveal undiagnosed problems? Is there the risk that only evident dental problems are addressed, while craniofacial growth problems remain underdiagnosed?

## 2. Materials and Methods

The authority that provided approval for this study was the North Emilia Ethics Committee of the University Hospital of Parma, with the resolution number 0000873 of 22 September 2020.

This retrospective study was carried out at the Dental Clinic of the Hospital of Parma (Italy), as part of the regional program of dental care. In the Italian public health system, everyone may be addressed for an orthodontic consultation, while clinical treatment is limited to specific weak groups of the population. Data were collected from a total sample of 540 consecutive patients referred to the Dental Clinic from June 2016 to December 2019; 40 were excluded because some data were incomplete. The final sample included 500 Caucasian patients (mean age 12.3 years, median 10.4 years). The indication for an orthodontic screening was investigated by asking the patients (or their relatives) whom they had been referred by. Possible answers to this question were: (1) family doctor; (2) paediatrician; (3) dentist; (4) other facial specialist: maxillo-facial surgeons and plastic surgeons; (5) relative or friend. The reason for orthodontic consultation was then investigated to assess how much the aesthetic dental irregularity or skeletal facial disharmony affects patients’ choices to undergo an orthodontic visit [9,10]. Patients were asked to indicate whether the reason for the visit was related to a dental aesthetic problem or a facial dysmorphism, and possible answers to this question were: (1) dental aesthetic irregularity, (2) facial disharmony. In this study a single expert specialist in orthodontics performed all the examinations to avoid inter-operator bias. During the visit, the specialist collected both dental and anthropometric facial data to diagnose dental irregularities and facial disharmonies. These data were cross-referenced with those relating to the motivation for the referral to evaluate its correctness.

(A) Dental data:

In the present study, the authors analysed the presence of the most frequent reasons for dental aesthetic irregularity of the smile: (1) overjet and overbite, (2) crowding or spacing between the frontal teeth. If even one of these parameters had been abnormal, the patient would have been diagnosed as affected by dental aesthetic irregularity and, in the opposite case, as normal on an aesthetic dental point of view.

**Figure 1:**
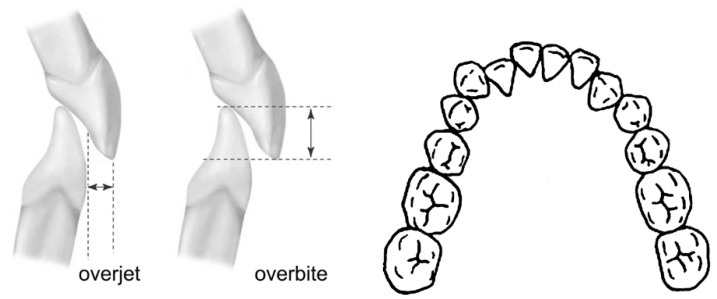
Overjet, overbite and crowding–spacing dental irregularities are frequently the main reasons for an orthodontic consultation.

Overjet and overbite: frontal teeth are immediately visible during speech and smile, and overjet/overbite (Figure 1) anomalies are often perceived as a cause of aesthetic discomfort. When the frontal teeth are protruding or in a cross-bite relation or when the bite is open or excessively deep, parents and doctors may be worried about the problem. In these cases, a specialistic visit is often requested.

Overjet—is defined as the distance between the incisal edge of the most labial maxillary central incisor and the labial surface of the corresponding mandibular incisor (or its extension perpendicular to the occlusal plane), measured to the nearest half millimetre using a metal ruler (Münchner Modell, No. 044-731-00, Dentaurum, Ispringen, Germany) parallel to the occlusal plane. A positive value is recorded if the upper incisor was ahead of the lower incisor and a negative value when the upper incisor is behind the lower incisor. The value considered as normal in the present study was 2 ± 1 mm. A distance of more than 3 or less than 1 mm was considered abnormal.

Overbite—is the vertical overlap of incisors, classified as positive if the incisors overlap vertically and as negative (open bite) if they are vertically separated. It was measured to the nearest half millimetre vertically from the incisal edge of the maxillary central incisor to the incisal edge of the corresponding mandibular incisor, on a line perpendicular to the occlusal plane (Figure 1). A positive value was recorded if the upper incisor was in a deep bite relation, and a negative value when the upper incisor was in open bite. The value considered as normal in the present study was 2 ± mm. An overlap of more than 3 or less than 1 mm was considered abnormal.

Crowding and spacing: crowding and spacing of the frontal teeth are also generally evident to parents and doctors and create aesthetic discomfort. Dental crowding is one of the more frequent motivations for orthodontic referral (Figure 1).

The scoring method for crowding was based on the Irregularity Index [22] used to assess the dental alignment of the lower incisors. 

Crowding: a score between 0 and 3 mm is generally considered as an anterior mild misalignment, whereas a score between 3 mm and 6 mm as a severe crowding. Perfect alignment from the mesial aspect of the left canine to the mesial aspect of the right canine is considered normal. The value considered as normal in the present study was ≥3. 

Spacing: Spacing between deciduous teeth is frequent and normal in deciduous dentition. In a mixed and permanent dentition, spacing between 0 and 3 mm is generally considered normal, spacing between 3 and 6 mm is perceived as moderate and spacing greater than 6 mm is considered severe. The value considered as normal in the present study was ≥6 in deciduous, ≥3 in mixed and 0 in permanent dentition.

(B) Anthropometric data:

Facial features are directly related to the harmony of the supporting maxillary bones. Particularly, the chin position is fundamental in creating sagittal or vertical disharmonies. The authors clinically analysed the position of the chin related to the face: (1) sagittal position, (2) vertical position, (3) symmetry. If even one of these parameters resulted to be abnormal, the patient would be diagnosed as affected by facial disharmony, and in the opposite case, as normal on a facial skeletal point of view.

(1) sagittal position

**Figure 2:**
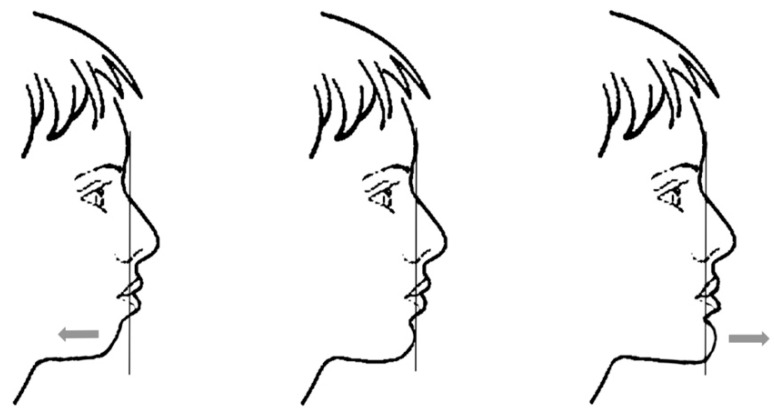
Sagittal position of the chin related to the vertical line from the glabella cutaneous point.

The sagittal position of the chin (Figure 2) was assessed by looking at the vertical line from the glabella point. Glabella is defined in the bone as a small, depressed area in the middle of the frontal bone, above the naso-frontal suture. In the facial surface, glabella is the midpoint between the super-ciliary arches. When the patient is in a “natural head position” (NHP) [23], the vertical line from the glabella can be easily recognized. In a normal chin sagittal position, this line touches the anterior limit of the chin (0 ± 4 mm.). When the measurement exceeds this limit one, can suspect an important skeletal class II or III situation. In this study, the authors considered abnormal a sagittal chin position <4 or >4 mm from this vertical line.

(2) vertical position

**Figure 3:**
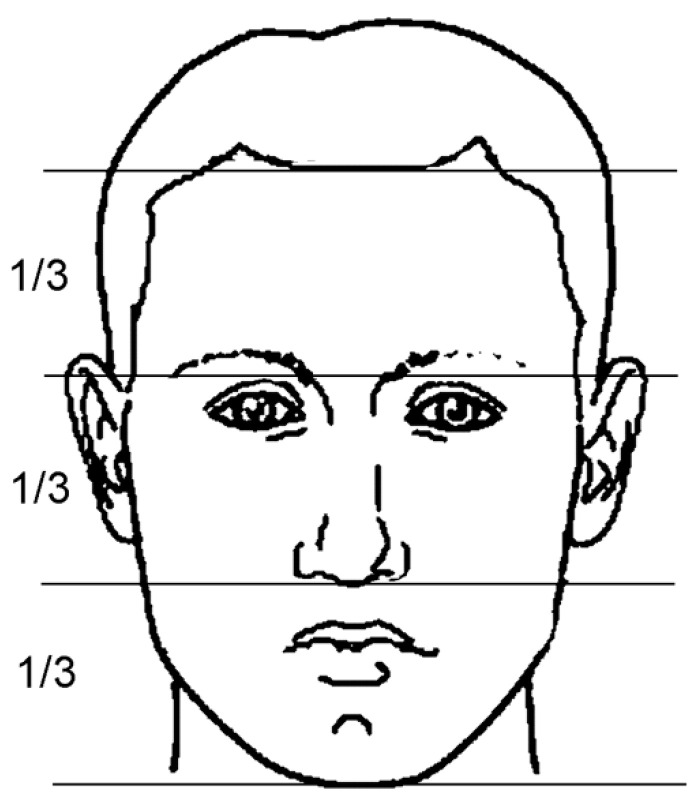
Vertical position of the chin related to the mid and upper third of the face.

The trichion (the hairline point), glabella, subnasal (the limit between the upper lip and the nasal contour) and cutaneous gnathion (lower limit of the chin) points divide the face into three parts (Figure 3). Artists well know that when these three parts are approximately equal, the face is in good vertical aesthetic harmony. When the lower third of the face is too long or too short, the face is unbalanced, and one can suspect an important vertical skeletal problem. In this study, the authors considered abnormal a vertical chin position <2 or >2 mm related to the middle third.

(3) symmetry

**Figure 4:**
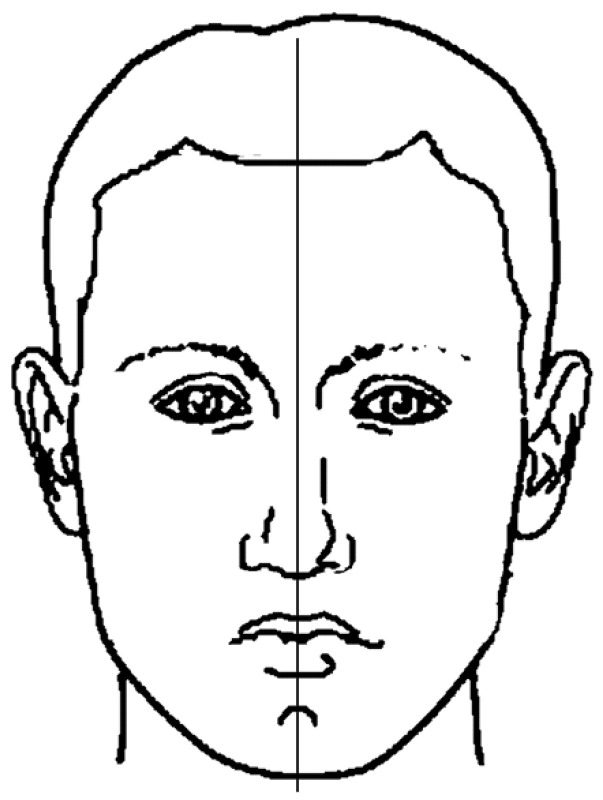
The symmetry axis of the face.

Looking at the face in a frontal view, it is possible to recognize chin deviation related to the midline of the face (Figure 4). The midline of the face is defined as the vertical line perpendicular to the bi-pupillary line and passing through the glabella point. Every face presents mild asymmetries, but in developing patients, the early diagnose of chin deviations is important in order to prevent a possible worsening during adolescence. When the deviation of the chin is over 2 mm, the patient should be examined to evaluate the need for treatment. In this study, the authors considered abnormal a sagittal chin deviation ≥2 mm.

## 3. Results

**Figure 5:**
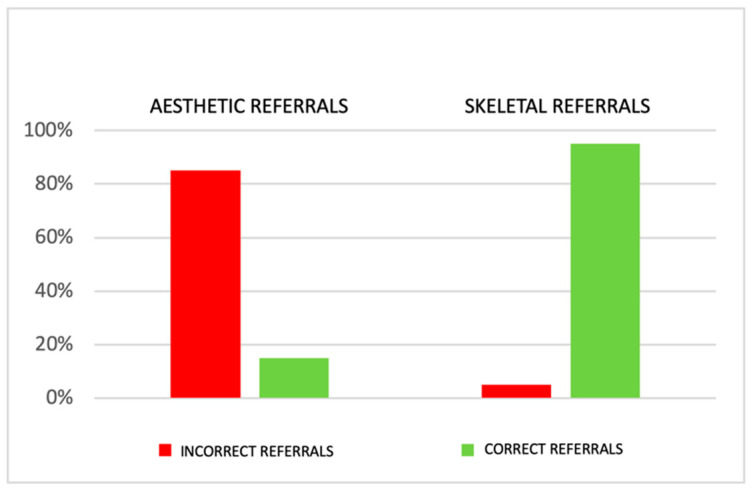
Overall sample. In total, 407 patients were referred for an aesthetic dental problem: 344 (85%) referrals were incorrect; 80 patients were referred for a skeletal problem: only in 4 (5%) cases, the referral was incorrect.

The sample of cases referred for aesthetic dental problems consisted of 407 patients. The orthodontic specialistic examination revealed skeletal problems in 344 cases, while in 63 cases, there were actually only dental aesthetic problems. The total sample of cases referred for skeletal problems included 80 patients. The orthodontic specialistic examination confirmed such problems in 76 cases, while only in 4 cases there were no such problems (Figure 5).

The referral for an orthodontics visit was made in 144 cases (28.8%) by the family dentist, followed by relatives or friends, who identified the need for a visit in 107 cases (21.4%), by paediatricians, who referred 94 cases (18.8%) and finally by family doctors, who referred 80 cases (16%) and other specialists, who presented 62 cases (12.4%). In 13 cases (2.6%), the referral source was unclear.

The main reasons for an orthodontic assessment were dental aesthetic problems (dental irregularities about overjet, overbite, crowding and spacing). In 412 cases (82.6% of the sample), the patient was referred for these reasons. Only in 81 cases (15.8% of the sample), the patients were addressed for facial disharmony. In seven cases (1.6%), the motivation was unclear.

The specialistic orthodontic examination revealed the following data. 

Regarding the dental analysis, in the frontal teeth, 280 patients (56%) presented an elevated amount of overjet. A negative overjet value was observed in 80 cases (16%), and a normal value in 133 (26.6%). Overbite was also elevated in the majority of cases: 294 (58.8%) patients presented a deep bite occlusion. A negative overbite value was observed in 117 (35.4%) cases, whereas a normal value only in 82 (16.2%). We can state that a large number of patients presented an elevate overjet and a deep bite occlusion. Space problems were also frequent. An excessive crowding was present in 259 (51.8%) patients, an excessive spacing in 44 (8.8%) patients, and normal spacing in 197 (39.4%) patients.

The orthodontist also made an anthropometric analysis. Regarding facial harmony, the authors state that also these irregularities were widely present in the sample. About the sagittal aspect of the chin, 226 (45.2%) cases presented a deficient chin projection and a class II aesthetical feature of the face. A protruded chin was present in 57 (11.4%) cases, thus creating the facial features of a class III deformity. A normal projection of the chin, accompanied by a normal profile, was observed in 213 (42.6%) cases. About the vertical aspect, 220 (44%) cases presented a vertical excess and a long face aesthetical feature. A deficient facial height was present in 91 (18.2%) cases, thus creating the features of a short face deformity. A normal vertical position of the chin, accompanied by a normal facial height, was observed in 186 (37.2%) cases. A good symmetry of the chin related to the midline of the face was diagnosed in the majority of cases 334 (66.8%). However, 165 (33%) cases presented an evident clinical chin deviation.

CROSS-REFERENCE OF THE DATA:

After the visit, data were cross-referred with those relating to the motivation for the referral.

**Figure 6:**
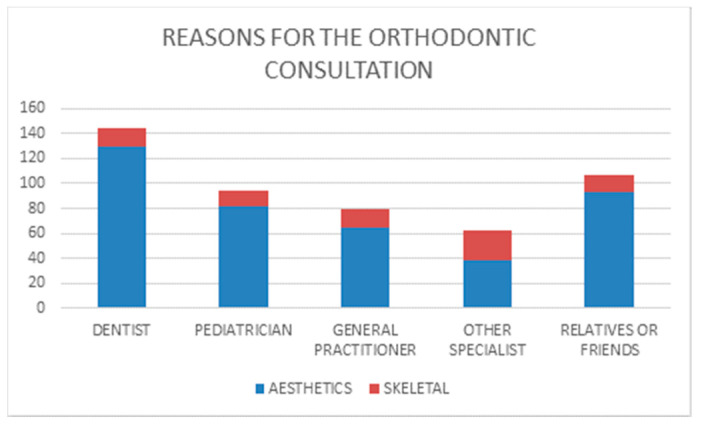
Reasons for the consultation.

**Figure 7:**
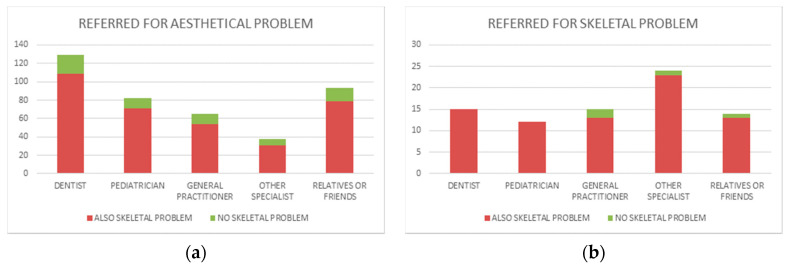
(**a**) In the group referred for aesthetic problems, several patients presented also undiagnosed skeletal disharmonies. (**b**) In the group referred for skeletal problems, almost all the diagnoses were correct.

Dentists referred 144 cases (Figure 6), 129 of which for dental aesthetic problems (90% of their referrals). In the group referred for aesthetic problems (Figure 7a), 109 cases (84.5%) presented also skeletal undiagnosed problems, whereas only 20 (15.5%) cases presented a good facial balance. They referred also 15 cases for skeletal problems (Figure 7b), and in this group, all patients were affected by these problems.

Relatives/friends referred 93 cases (Figure 6) for aesthetic problems (87% of their referrals). In the group of referrals for aesthetic problems (Figure 7a), 79 cases (85%) also presented skeletal undiagnosed problems, whereas only 14 (15%) cases presented a good facial balance. They referred also 14 cases for skeletal problems (Figure 7b), and in this group, only 1 case was not affected by these problems.

Paediatricians referred 94 cases (Figure 6), 82 of which for aesthetic problems (87% of their referrals). In the group of referrals for aesthetic problems (Figure 7a), 71 cases (86.6%) also presented skeletal undiagnosed problems, whereas only 11 (13.4%) cases presented a good facial balance. They referred also 12 cases for skeletal problems (Figure 7b), and in this group, all patients were affected by these problems.

General practitioners referred 80 cases (Figure 6), 65 of which for aesthetic problems (81% of their referrals). In the group of referrals for aesthetic problem (Figure 7a), 54 cases (83%) also presented skeletal undiagnosed problems, whereas only 11 (16%) cases presented a good facial balance. They referred also 15 cases for skeletal problems (Figure 7b), and in this group, only 2 cases were not affected by these problems.

Other specialists referred 62 cases (Figure 6), 38 of which for aesthetic problems (61% of their referrals). In the group of referrals for aesthetic problems (Figure 7a), 31 cases (86.5%) also presented skeletal undiagnosed problems, whereas 7 (18.4%) cases presented a good facial balance. They referred also 24 cases for skeletal problems (Figure 7b), and in this group, only 1 case was not affected by these problems.

## 4. Discussion

In a modern society, a regular dentition and an attractive smile are very important in improving self-esteem and social acceptance. The request for aesthetic improvement is therefore logical in our patients. When a dental irregularity is evident, doctors and relatives are justified in contacting a specialist in orthodontics. However, during facial growth, recognizing disharmonies in bone development is even more important. As R. M. Ricketts stated, “the early you treat, the more the face adapts to your ideal; the late you treat, the more you have to adapt to the face” several skeletal problems are only treatable in early age. This survey provides an estimate of the clinical dental and anthropometric features of an Italian population sample referred to the public health service and focuses on the analysis of the reasons for the referral to the orthodontic consultation. The first question concerned who asked for the orthodontic visit. In our sample, dentists and relatives/friends were the main source of the referrals. In addition, paediatricians referred several cases, and paediatricians and dentists together referred approximately one-half of the cases. 

This finding was expected, because these doctors are the first in contact with young patients. The authors believe that, for this reason, these professionals should be very precise in the early diagnosis of malformations. In the last position for referrals, we found other facial specialists (as maxillo-facial or plastic surgeons).

The second question concerned the main reasons for referral. We can state that aesthetic dental problems were the main reason for all of the five subsamples, but maxillo-facial and plastic surgeons frequently referred cases for skeletal disharmonies. It is not surprising that relatives and general practitioners identify almost only aesthetic problems, as they are not trained in analysing the harmony of the face, but dentists and paediatricians revealed surprisingly poor attention for the skeletal facial harmony. Maxillo-facial and plastic surgeons revealed, on the contrary, high attention for the facial skeletal harmony. These specialists are well trained in identifying facial deformities, but unfortunately, they are not so frequently in touch with young patients.

By looking at the subsample of patients only referred for aesthetical problems, we can state that the wide majority of cases was also affected by various skeletal disharmonies. Only few patients referred for dental disharmonies presented a good skeletal facial growth and development. This error was made by all subjects who referred cases in the same measure of about 80%. These data answer the last question about the risk that only evident dental problems are addressed, while craniofacial growth problems remain underdiagnosed. The perception of skeletal abnormalities such as asymmetry or discrepancies of the bone bases in the sagittal or vertical direction was significantly lower, thus suggesting a lack of awareness of these issues, most likely due to poor knowledge. Among the undiagnosed malformations, the authors found 103 class III patients, of which 42 were also affected by facial asymmetry, and 26 by long face and high-angle facial growth. It is well known that these skeletal problems are to be immediately diagnosed because they worsen with body development. There were also 154 undiagnosed cases of class II mandibular deficiency, and also these patients need early therapy. Our observations suggest that a large number of children suffering from facial skeletal disharmonies would not have been referred to the visit if they had not had associated dental aesthetic problems. These data suggest the need to increase the training of dentists and paediatricians in the early diagnosis of facial skeletal disharmonies, since they are often progressively worsening with development. On the contrary, when a patient was addressed for a skeletal facial disharmony, the correctness of the indication was very frequent. Especially, dentists and paediatricians were very accurate; they never referred a dysmorphic patient without a correct reason. Probably, the most severe facial malformations do not mislead those who recommend an orthodontic visit, while minor ones are not even recognized by doctors who are usually in touch with children: anyway, a large number of situations that may worsen with child growth are not reported.

## 5. Conclusions

In the northern Italy population, the risk that only evident dental problems are addressed to an orthodontic consultation, while craniofacial growth problems remain underdiagnosed, is confirmed by the present study. The medical figures who take care of young patients were shown to pay poor attention to the analysis of facial malformations, not very differently from general practitioners or even parents. The authors emphasise the importance of increasing the specific training of these medical figures to improve the early diagnosis of facial malformations.

## Data Availability

The data presented in this study are available on request from the corresponding author. The data are not publicly available due to ethical and privacy restrictions.
